# Aquaponic and Hydroponic Solutions Modulate NaCl-Induced Stress in Drug-Type *Cannabis sativa* L.

**DOI:** 10.3389/fpls.2020.01169

**Published:** 2020-08-05

**Authors:** Brandon Yep, Nigel V. Gale, Youbin Zheng

**Affiliations:** ^1^School of Environmental Sciences, University of Guelph, Guelph, ON, Canada; ^2^Faculty of Forestry, University of Toronto, Toronto, ON, Canada

**Keywords:** aquaponics, cannabis, cannabinoids, salt stress, salt tolerance, solution culture, hydroponics, marijuana

## Abstract

The effects of salt-induced stress in drug-type *Cannabis sativa* L. (*C. sativa*), a crop with increasing global importance, are almost entirely unknown. In an indoor controlled factorial experiment involving a type-II chemovar (i.e., one which produces Δ^9^-tetrahydrocannabinolic acid ~THCA and cannabidiolic acid ~ CBDA), the effects of increasing NaCl concentrations (1–40 mM) was tested in hydroponic and aquaponic solutions during the flowering stage. Growth parameters (height, canopy volume), plant physiology (chlorophyll content, leaf-gas exchange, chlorophyll fluorescence, and water use efficiency), and solution physicochemical properties (pH, EC, and nutrients) was measured throughout the experiment. Upon maturation of inflorescences, plants were harvested and yield (dry inflorescence biomass) and inflorescence potency (mass-based concentration of cannabinoids) was determined. It was found that cannabinoids decreased linearly with increasing NaCl concentration: -0.026 and -0.037% THCA·mM NaCl^-1^ for aquaponic and hydroponic solutions, respectively. The growth and physiological responses to NaCl in hydroponic—but not the aquaponic solution—became negatively affected at 40 mM. The mechanisms of aquaponic solution which allow this potential enhanced NaCl tolerance is worthy of future investigation. Commercial cultivation involving the use of hydroponic solution should carefully monitor NaCl concentrations, so that they do not exceed the phytotoxic concentration of 40 mM found here; and are aware that NaCl in excess of 5 mM may decrease yield and potency. Additional research investigating cultivar- and rootzone-specific responses to salt-induced stress is needed.

## Introduction

Rootzone salinity (NaCl) is a predominant stress factor that poses three main problems to glycophyte conventional field crops. Soil water potential is first reduced by salt-induced drought, followed by toxicity from uptake of Cl^-^ and Na^+^, and finally from perturbations in nutrient stoichiometry (Munns, 2002; Gupta and Huang, 2014; Isayenkov and Maathuis, 2019). Areas with sodic or non-arable soils are increasingly using recirculating solution systems as an alternative agriculture system for water conservation and rootzone optimization (Rafiee and Saad, 2006; Oladimeji et al., 2018); however, these systems are not liberated from rootzone salinity. Plants cultivated in recirculating solution systems can accumulate Na^+^ and Cl^-^ at concentrations found to be damaging for most greenhouse crops (e.g., 3–21 mM NaCl) (Beauchamp et al., 2018; Goddek and Vermeulen, 2018). As water is reused in recirculating systems, un-absorbed ions can accumulate to concentrations which create salt stress (i.e., 5–10 mM NaCl; Sonneveld et al., 1999; Neocleous and Savvas, 2017), or disrupt the uptake of other dissolved mineral nutrients. Na^+^ and Cl^-^ often accumulate in these systems as a result of high Na^+^/Cl^-^ containing source water and/or fertilizers, paired with low Na^+^/Cl^-^ requiring crops (Stanghellini et al., 2005).

The salinity dilemma, that is that salts cause osmotic and water stress but also are osmoticum that supports cell osmotic pressure (Gupta and Huang, 2014; Isayenkov and Maathuis, 2019) is expected to differ between aqueous solution types as a function of the plant extracellular physicochemical properties and microbiome. Previous studies investigating the effects of dissolved NaCl in solution, have primarily used a modified Hoagland solution [i.e., soluble mineral fertilizer solution, with 12 N, 2 P, and 6 K (mM)]. An alternative solution that has not been well investigated for NaCl experiments, is aquaponic solution. Aquaponics is an emerging form of controlled environment agriculture, utilizing the wastes from aquaculture as a source of nutrients for crop production. This system has gained recent commercial popularity as a sustainable system capable of producing both plants and fish, while concomitantly recycling resources (i.e., water and nutrients) (Yep and Zheng, 2019). Due to the high water-use efficiency of aquaponics (consumes 0.3–5% of total system solution per day; Rakocy et al., 2010; Maucieri et al., 2018), solution is recirculated longer than most recirculating hydroponic systems, potentially resulting in an accumulation of Na^+^ and Cl^-^, as observed by Yep (2020). The chemical constituents of aquaponics solution is also markedly different than most hydroponic solutions, namely reduced K:N and P:N (Seawright et al., 1998; Tyson et al., 2011; Roosta, 2014; Nozzi et al., 2018). Physically, aquaponics contains appreciable organic matter that is subject to mineralization, supplying available nutrients. This organic matter, and to some extent other particulates, have inherent negative pressures (Asadi et al., 2009) acting also as osmoticum to improve water status and reduce “drought” stress in saline solutions (Isayenkov and Maathuis, 2019). Aquaponic systems also depart from conventional hydroponic solution biologically, as most comprise a rich microbiome that includes plant growth promoting microbes (PGPM) found to increase resistance to infection from the root pathogens *Pythium* and *Fusarium* spp. (Gravel et al., 2015). Furthermore, nitrifying bacteria in aquaponic solution provides a steady supply of NO_3_^-^ from organic sources of NH_4_ (Wongkiew et al., 2017). The continous supply of NO_3_^-^ may alleviate the oxidative inducing Cl^-^ stress assocaited with NaCl, given that increasing NO_3_^-^ supply has shown to reduce cellular Cl^-^ toxicity by lowering NO_3_^-^ from being outcompeted in the rhizosphere (Guo et al., 2017). Many PGPM genera, such as *Bacillus*, *Streptomyces, and Pseudomonas*, can alleviate salt stress in soils through a variety of other mechanisms; however, this has not been investigated in aquaponic solution (Dodd and Pérez-Alfocea, 2012; Egamberdieva et al., 2019). It was expected that the concentration-response relationships of plants to NaCl will vary by solution physicochemical properties and microbiome; however, comparisons of NaCl-induced stress in contrasting solution types had not been made.

The effects on salinity on common greenhouse vegetable crops have been well investigated, and share a relatively common phytotoxicity threshold for NaCl in recirculating systems (5–10 mM) (Sonneveld et al., 1999; Shaheen et al., 2013; Neocleous and Savvas, 2017); however, effects of salinity on the increasingly important crop *Cannabis* sativa L. (herein referred to as *C. sativa*) has received minimal previous research attention. *C. sativa* is an annual herbaceous crop of increasing economic importance for a multitude of uses: hemp-type cultivars are a significant source of bast and woody fibers and drug-type cultivars produce a trove of secondary metabolites (namely Δ^9^-tetrahydrocannabinolic acid ~ THCA and cannabidiolic acid ~ CBDA) which are of significant medicinal value (Gonçalves et al., 2019). Hemp-type cultivars of *C. sativa* [those which produce < 0.3% THC according to Small et al. (1975)] demonstrate several mechanisms to enhance tolerance to NaCl (Liu et al., 2016; Guerriero et al., 2017). Hemp has shown some ability to tolerate NaCl in trials involving seed germination (> 70% germinating in 150 mM NaCl) (Hu et al., 2018) and seedling development (4.4% greater chlorophyll content at 100 mM NaCl compared to the control) (Hu et al., 2019). Some evidence suggests salt tolerance in hemp is associated with the upregulation of heat-shock proteins, genes associated with secondary wall and lignin biosynthesis, and most recently, aquaporins capable of regulating water transfer across the cell membrane, potentially improving water-stress in saline plant extracellular matrices (Guerriero et al., 2017; Guerriero et al., 2019). Another potential adaptation to salts in hemp is hyper-accumulation of Si in the bast-fiber cell walls, which alongside aquaporins, enhance water status (Guerriero et al., 2019). Si has shown to ameliorate the reduction in water use efficiency caused by salinity (Rios et al., 2017); however, salinity’s effect on water use efficiency has varied in the past research, sometimes resulting in it to increase (Chartzoulakis and Klapaki, 2000; Barbieri et al., 2012; Lovelli et al., 2012) or decrease (Shaheen et al., 2013) depending on species and the osmoticum creating salinity. Whether drug-type cultivars share similar relative tolerance to salinity is entirely unknown. Furthermore, effects of NaCl on *C. sativa* have not been investigated in more commercially employed soilless production systems, such as those with aqueous recirculating solution.

To determine the influence of NaCl on *C. sativa* growth, physiology, yield, and potency (secondary metabolite production), in a controlled environment, increasing concentrations of NaCl from 1 to 40 mM was applied in conventional hydroponic and aquaponic solutions during the flowering stage of growth. It was hypothesized that plants would have concentration-dependent responses to NaCl, predicting a trend of declining growth, physiological performance, yield, and potency as concentrations of NaCl increase. It was also expected that NaCl tolerance would vary by solution due to contrasting biogeochemistry between hydroponic and aquaponic solutions.

## Materials and Methods

### Plant Culture: Propagation and Environmental Conditions

The experiment was conducted at an indoor license holder of medical *C. sativa*, located in Ontario, Canada. The *C. sativa* cultivar “Nordle”, a type-II chemovar [having THCA > 0.3% and CBDA > 0.5% as classified by Small et al. (1975)], was used for the experiment. One hundred twenty plants were propagated by excising meristematic segments (i.e., cuttings, ~ 25 cm in length) from the terminal portions of uppermost canopy of a vegetative stock (mother) plant (grown under an 18 h photoperiod) and inserting these cuttings into J7 Hort. 42 x 43 mm peat pellets (Jiffy Products, Shippgan, NB, Canada) containing a powdered rooting hormone with the active ingredient, 0.1% indole-butyric acid (Stim-Root No. 1, Master Plant-Prod Inc., Brampton, Ontario Canada). Upon formation of root apical meristems (up to 10 days), the rooted cuttings were individually transplanted into 813 ml circular net pots with 0.5 x 1.5 cm perforations throughout the pot (height 8.5 cm; diameter_1_ 12.5 cm; diameter_2_ 9.5 cm), filled with a custom soilless substrate consisting of primarily peat and trace amounts of bagged top soil, compost, turface, and biochar. The sides of the pots were lined with opaque polystyrene to contain the substrate and prevent light from penetrating into solutions. Vegetative growth occurred for 21 days in a controlled room with the following conditions at the canopy-level: 24.1 ± 0.43°C (mean ± standard deviation), relative humidity of 70.2 ± 1.96%, and photosynthetically active radiation (PAR) of 350 ± 21.3 µmol·m^-2^·s^-1^. Sixty plants of uniform size were selected and moved into a flowering room where they were inserted into 8 L deep water culture opaque polystyrene buckets and exposed to a 12 h photoperiod. Dissolved oxygen was maintained in solution at a concentration of 7.8 ± 0.13 mg·L^-1^ – aerated by 2.54 cm oxygen stones. Plants were arranged at a density of six plants per square meter. For the first 11 days in the flowering stage [DFS – day(s) in the flowering stage], all plants were grown in a complete mineral fertilizer solution with an electroconductivity (EC) of 1.0 mS·cm^-1^ and a pH of 5.7 (MJ Bloom™ at 1 g·L^-1^, Master Plant-Prod Inc., Brampton, Ontario, Canada), to allow sufficient roots to develop into the solution. At 12 DFS, after a minimal root meristem length of 10 cm emerged from the exterior of the pot, plants were subjugated to their respective aqueous treatment solutions.

During the flowering stage, light was supplied from light emitting diodes (Pro650, Lumigrow Inc. Emeryville, California, USA) at a 2:1:7 ratio of blue, green, and red wavelengths which provided PAR at 655 ± 68.7 µmol·m^-2^·s^-1^ at a 12 h photoperiod. During the light period conditions at the canopy level were: 29.7 ± 1.37°C, relative humidity of 41.8 ± 3.69% and CO_2_ concentration of 436 ± 38.4 ppm. During the dark period conditions at the canopy level were: 26.2 ± 0.98°C, relative humidity of 44.0 ± 4.59% and CO_2_ concentration of 479 ± 42.2 ppm.

### Experimental Design and Treatments

The experiment was a completely randomized design with two factors: solution type and NaCl concentration. Solution was either hydroponic or aquaponic. Each solution type had five different concentrations of NaCl. Hydroponic solutions had NaCl concentrations of (in mM) 1, 5, 10, 20, and 40; and aquaponic solutions had NaCl concentrations of (in mM) 4, 8, 10, 20, and 40. Each solution and NaCl concentration combination was replicated six times, with each replicate being an individual plant grown in one of the 10 NaCl and solution combinations (2 solution x 5 concentrations x 6 replicates = 60 plants). All solutions were replaced with 8 L of fresh treatment-based solution when any of the following occurred for any single plant: pH changed by more than 0.5, more than of 50% of solution was depleted, or EC changed by more than 20% in any given solution. Solution was also changed for all plants every seven days, if none of the prior criteria was met. Each week plant locations were randomized to mitigate minor environmental variation in the air (i.e., varying light intensity, airflow, and temperature).

Hydroponic solution was prepared by mixing reverse osmosis filtered water with “Plant-Prod MJ™”, a commonly used commercial fertilizer for *C. sativa* (Master Plant-Prod Inc., Brampton, Ontario, Canada), according to the manufacturer’s recommendations. The nutrient concentrations and EC for the primary hydroponic solution and aquaponic solution are presented in **Table 1**. To prepare an aquaponic solution with lower Na^+^ and Cl^-^ concentrations than the source solution (7.7 mM Na^+^ and 7.8 mM Cl^-^), aquaponic solution was mixed 1:1 with reverse osmosis filtered water. A fertilizer containing (in mM) 2.7 N, 1.6 P, and 1.6 K (MJ Bloom and MJ Cal Kick together, each at 0.38 g·L^-1^, Master Plant-Prod Inc., Brampton, Ontario, Canada) was also added at one third concentration of the recommended rate to compensate for the dilution based on EC. Aquaponic solution was sourced from the deep water culture basin of a mature (five year operating) commercial coupled aquaponic system (Nelson and Pade^®^, Montello, Wisconsin, USA) stocked with 1,189 adult *Oreochromis niloticus* (Nile Tilapia) each approximately 1.0 kg, 11 months old and stocked at a density of 98 fish·m^-3^. Fish were fed 1,600 g of 4 mm “Floating Feed” (Corey Aqauafeeds, Fredericton, New Brunswick, Canada) per day. The system was a single loop recirculating system with the same design as The University of the Virgin Islands presented in Rakocy (2012). The conversion of organic nutrients into plant available inorganic ionic nutrients in the mineralization tanks and bioreactor (mineralization process) was fully functioning and at equilibrium, based on nutrient concentration consistency measured in the preceding six months (data not shown). The entire system held 49,000 L of solution, comprising four individual 2.4 m x 14.6 m (35 m^2^) deep water culture basins to grow plants. Aquaponic solution was maintained at 23 ± 0.1°C and a dissolved oxygen concentration of 11 ± 1.7 mg·L^-1^. Aquaponic and hydroponic solutions were adjusted to a pH of 5.70 ± 0.025 using H_3_PO_4_. NaCl treatments were applied by mixing pre-weighed laboratory grade inorganic NaCl (Fisher Scientific, New Jersey, USA) to each individual plant solution upon change. Treatment-appropriate Na^+^ concentration for each solution was verified for each solution using a portable ion selective electrode meter (LAQUAtwin-: Na-11, Horiba Scientific, Kyoto, Japan).

**Table 1:** Plant essential mineral nutrient concentrations, ratios, and EC of two solution types (aquaponic and hydroponic).

Mineral nutrient	Aquaponic	Hydroponic
NO_3_-N	8.71	12.78
NH_4_-N	2.06	5.20
P (H_2_PO_4_)	1.88 (2.39)	3.05 (3.91)
K	2.38	5.14
Mg	1.32	0.58
Ca	3.72	3.42
SO_4_	1.78	0.94
B	0.01	0.021
Cu	0.003	0.008
Fe	0.017	0.021
Mn	0.004	0.009
Zn	0.006	0.008
EC	1.94 ± 0.078	1.80 ± 0.072
N:P	2.59	2.67
N:K	1.62	1.25
N:Mg	4.70	17.9
N:Ca	1.01	1.84
P:K	0.63	0.47
P:Mg	1.82	6.70
P:Ca	0.39	0.69
K:Mg	2.90	14.3
K:Ca	0.62	1.47

Mineral nutrient values are presented as mM, ratios (x:y) are presented as a ratio of the nutrients expressed in mg·L^-1^ and EC is presented as mean ± standard deviation (S·m^-1^) over the course of the experiment.

### Plant Growth and Physiological Performance Measurements

Each week, plant height, growth index, number of branches, and leaf chlorophyll content index (CCI) was measured. Plant height was measured from the base of the substrate to the apical meristem to the nearest cm. Growth index was measured using plant height, and two perpendicular canopy width measurements, using markers on the pot as reference points and calculated according to Ruter (1992) [(height × width_1_ × width_2_) ÷ 300]. Growth index has been previously used as a canopy volume metric for *C. sativa* by Caplan et al. (2017). CCI was measured on the center leaflet of the most recently expanded leaf by taking triplicate measurements using a portable chlorophyll content meter (CCM-200 Chlorophyll Concentration Meter, Opti-Sciences Inc., Hudson, New Hampshire, USA).

At 21, 35, and 56 DFS, leaf net CO_2_ assimilation rate (A), net CO_2_ assimilation rate at light saturation (A_sat_) and stomatal conductance (*g_s_*) was measured on the center leaflet of the newest fully matured leaf of each plant, using a portable photosynthesis machine (LI-6400XT, LI-COR Biosciences, Lincoln, NE). The conditions of the chamber were maintained at: CO_2_ of 500 ppm, temperature of 28.0°C, ambient relative humidity (41.8 ± 3.69%), a flow rate of 500 µmol·s^-1^ and PAR at 350 µmol·m^-2^·s^-1^ and 1,500 µmol·m^-2^·s^-1^ for A, and A_sat_, respectively. A PAR of 1,500 µmol·m^-2^·s^-1^ was used for A_sat_ as it was predetermined by measuring leaf light response curves on the experimental plants (data not shown) and a PAR of 350 µmol·m^-2^·s^-1^ was used for A as it was the lowest measured intensity at the canopy height.

At 21, 35, and 56 DFS chlorophyll fluorescence parameter Fv/Fm using a portable fluorometer (FluorPen FP 100, Photon Systems Instruments, Drasov, Czech Republic) was measured on the center leaflet of the most recently expanded leaf of each plant. Fv/Fm was measured by dark adapting leaf tissue for 20 min using detachable leaf clips. The fluorometer was then attached to the leaf clips and fluorescence was measured before and after the fluorometer emitted a 2,100 µmol·m^-2^·s^-1^ light pulse. Water use efficiency, in terms of dry inflorescence (at 13% moisture content) produced per L of solution absorbed over the experiment, was calculated as: dry inflorence biomass (g) ÷ total solution uptake (L).

### Solution Physicochemical Properties

Solution uptake and changes in pH and EC were measured at every solution exchange. Solution uptake was calculated using the height differences of the solution in the container right after the change and before the next change of nutrient solution. Solution loss due to evaporation was minimal but was standardized through by measuring loss in aerated solution without a plant, over seven days. Na^+^, Ca^2+^, and K^+^ removal (measured in mg) was calculated by subtracting the total element weight three days after solution exchange, from the initial total element weight. Element weights were calculated by multiplying the solution volume (L) by the concentrations of the element (mg·L^-1^)—using portable ion selective electrode meters (LAQUAtwin-: Na-11, K-11, Ca-11, Horiba Scientific, Kyoto, Japan). Solution pH and EC was measured at each solution exchange with a portable pH/EC meter (W-35631-00 Portable Waterproof pH/Con 300 Meter, Oakton Instruments, Vernon Hills, Illinois, USA).

Nutrient removal was measured at 35 DFS, a time point previously shown to be the time at which vegetative growth (i.e., canopy volume) ceases and only reproductive growth continues under a 12 h photoperiod (Yep, 2020). Nutrient removal was determined by taking three solution samples from each treatment three days after new solution was applied and analyzing H_2_PO_4_, NH_4_-N, NO_3_-N, SO_4_, B, Ca, Cu, Fe, K, Mg, Mn, Mo, Na, P, Si, and Zn, at an independent commercial laboratory (A&L Canada Laboratories Inc., London, Ontario, Canada). Metals were analyzed with inductively coupled plasma optical emission spectrometry. For each nutrient analyzed, the solution volume in L (V_2_) and nutrient concentration in mg·L^-1^ (N_2_) after three days, was compared to initial nutrient concentrations (N_1_) in the stock solution and the initial solution volume (V_1_), to calculate nutrient removal through the following equation: Nutrient removal = (*V*_1_ × *N*_1_) – (*V*_2_ × *N*_2_).

### Inflorescence Yield and Potency

At 68 DFS plants were harvested and inflorescence biomass (yield) was separated by manually trimming entire inflorescences from the plant. Inflorescences were wet weighed and let to dry in paper bags in a drying room maintained at 15 ± 0.8°C and a relative humidity of 40 ± 2.9% for seven days. Once inflorescence biomass reached an average moisture content of 13 ± 2.3% determined through measuring moisture content of composite treatment samples at the 5^th^, 6^th^, and 7^th^ days in the drying room through thermo-gravimetric loss (Mettler Toldeo Halogen Moisture Balance, Mettler-Toldeo AG, Greifensee, Switzerland), the dry weight was measured and adjusted to 13% moisture content based on the measured moisture content of the treatment sample, following the current commercial *C. sativa* industry’s common practice. After drying, three 10 g composite inflorescence biomass samples from the apical meristems of each treatment were analyzed for concentrations of Δ^9^-tetrahydrocannabinol (THC), cannabidiol (CBD), cannabinol (CBN), cannabigerol (CBG), cannabichromene (CBC), tetrahydrocannabivarin (THCV), cannabidivarin (CBDV), the acid forms of the previous cannabinoids, cannabigerovarin acid (CBGVA) and total terpenoids at a commercial cannabis testing laboratory (Anandia Laboratories, Vancouver, British Columbia, Canada). Cannabinoids were analyzed using ultra-high-performance liquid chromatography and mass spectrometry detection, while terpenoids were analyzed using gas chromatography and mass spectrometry detection. The mean cannabinoid content (amount) per plant was calculated as: dry inflorescence biomass (g·plant^-1^) × mean cannabinoid concentration (%).

### Statistics

To test for significant NaCl-effects on *C*. *sativa* growth, physiology, yield, potency, and solution physiochemical properties, data for each solution was analyzed separately using JMP Statistical Discovery Version 14.0 (SAS Institute Inc., Cary, NC). For all tests, a Type-I error rate of α = 0.05 was used to determine the significance of the results. For solution physiochemical properties, growth and physiological performance parameters measured through time—i.e., Na^+^, K^+^, Ca^2+^ removal, plant height, canopy growth index, number of branches, CCI, leaf-gas exchange parameters and Fv/Fm, the effect of the NaCl (fixed-factor), time (fixed-factor) and NaCl through time (fixed-factor) was tested with a repeated-measures ANOVA. Since data were non-parametric, the models were fitted with restricted maximum likelihood (REML) standard least-squares personality tests as per SAS Institute Inc. (2018). The denominator degrees of freedom in these analyses were adjusted using the Kackar-Harville correction (Kackar and Harville, 1984; Kenward and Roger, 1997).

The effect of NaCl on dry inflorescence biomass (yield), cannabinoid concentrations (potency), and nutrient removal at 35 DFS were tested with linear regressions. Linear models were determined to be best fit models by comparing adjusted R^2^ values of all models that were significant for each trait (i.e., linear, quadratic, and cubic). To test that the data met the assumptions of each separate linear model, Brown-Forsythe tests were performed on the variance of the model’s residual values above and below the median predicated values to determine homoscedasticity; and normality was tested by Shapiro-Wilk W Tests on the distribution of the model’s residual data. To determine differences in dry inflorescence biomass, the effect of NaCl on dry inflorescence biomass was also tested with a one-way ANOVA, followed by Tukey’s-HSD (honestly significant difference) pairwise comparisons of NaCl treatments if significance was detected in the model.

To determine the mechanisms responsible for observed yield responses to NaCl, bivariate analyses were conducted determining the coefficients of Pearson correlations between physiological parameters (A, Fv/Fm, *g*_s_) and dry inflorescence biomass.

## Results

### NaCl Influences Plant Physiology and Growth

**Figure 1:**
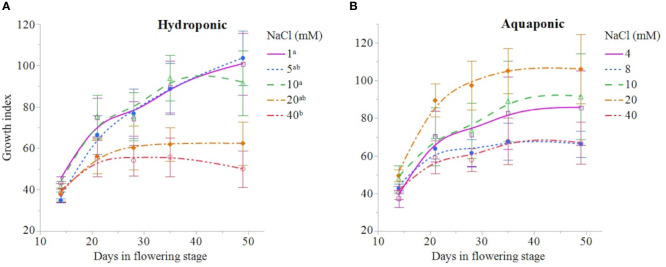
*C. sativa* growth index [(height × width_1_ × width_2_) ÷ 300] throughout the flowering stage (time) for plants grown in hydroponic **(A)** and aquaponic **(B)** solution with different NaCl concentrations. Data points are mean ± standard error (n = 6). NaCl concentrations with differing superscript letters (i.e., ^a,b^) indicate signficant differences according to contrast statements (F-tests) comparing all time points at *P* < 0.05.

**Figure 2:**
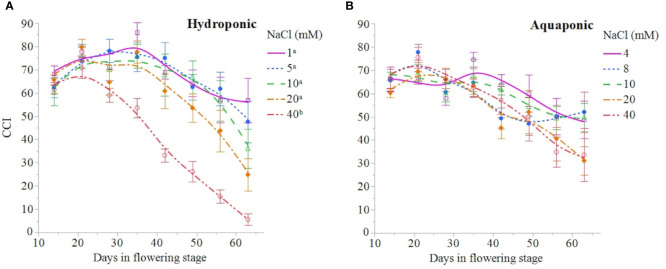
*C. sativa* leaf chlorophyll content index (CCI) throughout the flowering stage (time) for plants grown in hydroponic solution **(A)** and aquaponic **(B)** with different NaCl concentrations. Data points are mean ± standard error (n = 6). NaCl concentrations with differing superscript letters (i.e., ^a,b^) indicate signficant differences according to contrast statements (F-tests) comparing all time points at *P* < 0.0001.

**Figure 3:**
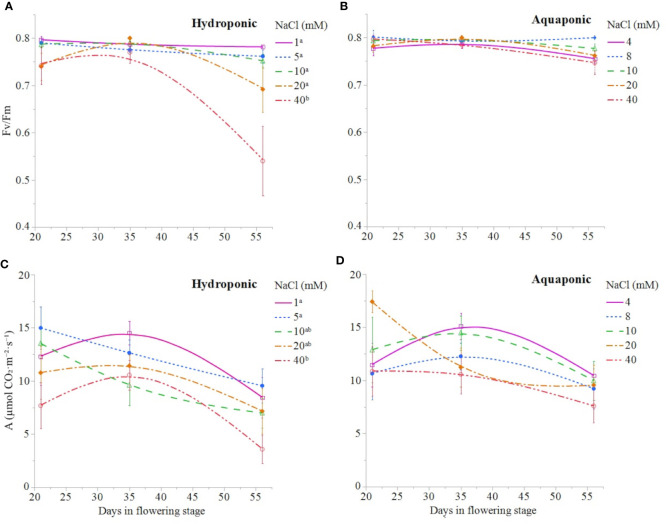
*C. sativa* leaf fluorescence parameter Fv/Fm and leaf net CO_2_ assimilation throughout the the flowering stage (time) for plants grown in hydroponic **(A, C**, respectively) and aquaponic **(B, D**, respectively) solution with different NaCl concentrations. Data points are mean ± standard error (n = 6). NaCl concentrations with differing superscript letters (i.e., ^a,b^) indicate signficant differences according to contrast statements (F-tests) comparing all time points at *P ≤* 0.0133 for Fv/Fm values and at *P* ≤ 0.0096 for A values.

NaCl concentration affected the growth and physiology of *C. sativa* plants grown in hydroponic solution but not those grown in the aquaponic solution. Most parameters that were measured over time had reduced values in plants grown in 40 mM NaCl hydroponic solution. NaCl affected growth index, CCI, Fv/Fm, and A over time in hydroponic solution, but not in aquaponic solution (**Table 2**). Plants grown in hydroponic solution at 40 mM NaCl had reduced growth index over time compared to plants grown at 1 and 10 mM NaCl hydroponic solutions, while NaCl did not have an effect on plants grown in aquaponic solution (**Figures 1A, B****)**. Plants grown in 40 mM NaCl hydroponic solution also had reduced CCI overtime compared to all other NaCl concentrations, while plants grown in aquaponic solution were not affected (**Figures 2A, B****)**. The photosynthetic rate and maximum potential quantum efficiency of photosystem II were impaired when NaCl exceeded 40 mM as evident through significant differences over time in Fv/Fm and A measurements; with a noticeable decline at 56 DFS (**Figures 3A, C****)**. Alternatively, plants grown in aquaponic solution did not responded to NaCl concentrations, in regard to leaf-level physiological parameters (**Figures 3B, D****)**. Results from the correlations between dry inflorescence biomass and physiological parameters at 56 DFS for plants in hydroponic solution, reveal positive relationships: A (r = 0.52; *P* = 0.0032), *g_s_* (r = 0.54; *P* = 0.0019), and Fv/Fm (r = 0.58; *P* = 0.0007).

**Table 2:** *C. sativa* growth and physiology performance parameters response to NaCl concentration in hydroponic and aquaponic solution, throughout the flowering stage (time), as determined by a repeated measures ANOVA.

Parameter	Aquaponic/Hydroponic	SE	df (N,D)	F-ratio	*P*
Growth index**	Hydroponic	120.342	16, 100	2.602	0.0020
	Aquaponic	226.320	16, 100	0.971	0.4935
CCI**	Hydroponic	26.039	28, 175	2.579	<.0001
	Aquaponic	25.198	28, 175	1.394	0.1028
Fv/Fm**	Hydroponic	0.000740	8, 50	3.469	0.0030
	Aquaponic	0.000147	8, 50	0.981	0.4618
A*	Hydroponic	3.168	8, 50	3.129	<.0324
	Aquaponic	3.806	8, 50	0.918	0.4691

*Refers to NaCl-effect, **refers to NaCl*time-effect, growth index is canopy volume·300^-1^, CCI is chlorophyll content index, A is leaf net CO_2_ assimilation, SE is total standard error (of residual and plant ID), and df (N,D) is degrees of freedom (numerator, denominator).

### Effects of NaCl on Inflorescence and Potency

**Figure 4:**
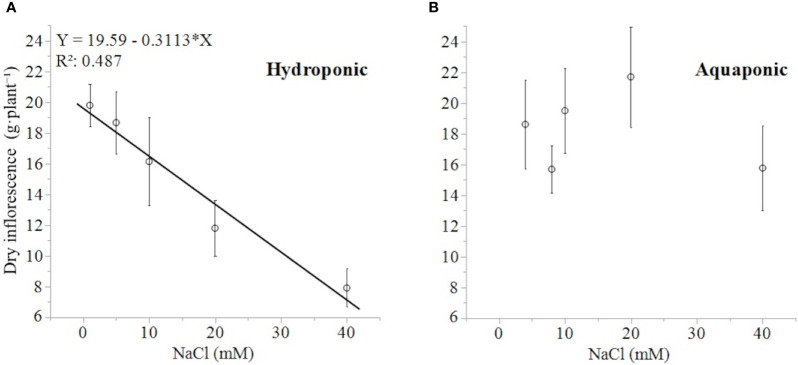
*C. sativa* dry inflorescence biomass (at a moisture content corrected to 13%) in response to increasing NaCl concentrations in hydroponic **(A)** and aquaponic **(B)** solution. Where markers are means with standard error mean bars (n = 6) and line is best fit regression relationship at *P* < 0.0001.

The response of dry inflorescence biomass was concentration-dependent, decreasing linearly with increasing NaCl concentrations (-0.31 g·mM NaCl^-1^), in plants grown in hydroponic but not in aquaponic solution (**Figures 4A, B****)**. Plants grown in hydroponic solution had the highest dry inflorescence biomass in the 1 mM NaCl treatment with 19.8 ± 1.38 g·plant^-1^ (mean ± standard error of mean), and the lowest dry inflorescence biomass in 40 mM NaCl treatment, with 7.9 ± 1.24 g·plant^-1^. Plants in the 40 mM NaCl treatment had 150, 136, and 104% lower dry inflorescence biomass than plants in the 1, 5, and 10 mM treatments, respectively [NaCl effect: F(4,29) = 6.41; *P* < 0.0011]. Alternatively, there was no relationship between the dry inflorescence biomass of plants grown in aquaponic solution to NaCl concentration. Plants grown in aquaponic solution had a mean dry inflorescence biomass of 18.3 ± 6.56 g·plant^-1^.

**Figure 5:**
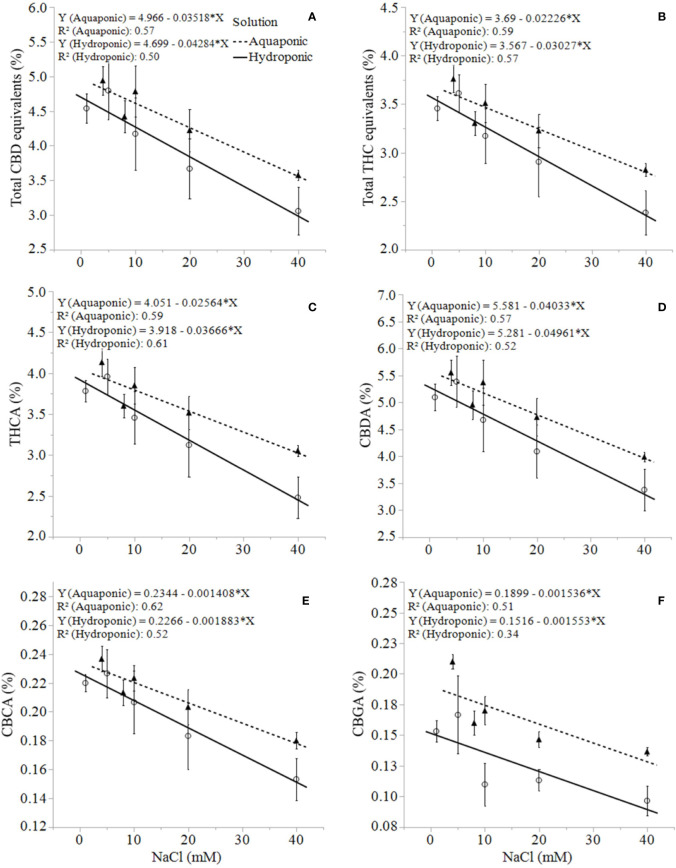
*C. sativa* dry inflorescence total CBD equivalents **(A)**, total THC equivalents **(B)**, THCA **(C)**, CBDA **(D)**, CBCA **(E)**, and CBGA **(F)** concentrations at maturity in response to increasing NaCl concentrations in hydroponic and aquaponic solutions. Where markers are means with standard error mean bars (n = 3) and lines are best fit regression relationships with *P* ≤ 0.0224.

**Figure 6:**
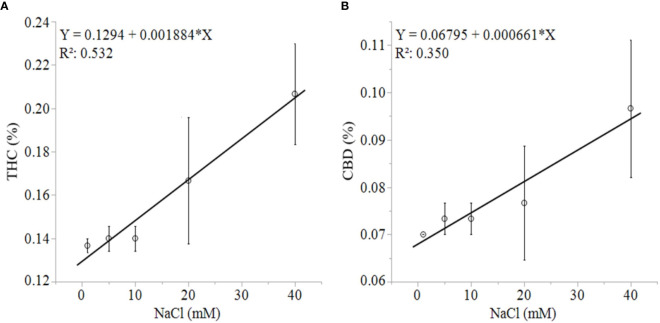
*C. sativa* dry inflorescence THC **(A)** and CBD **(B)** concentrations in response to increasing NaCl concentrations in hydroponic solution. Data points are means with standard error mean bars (n =3) and lines are best fit regression relationships with *P ≤* 0.0202.

Unlike growth, physiology, and biomass responses, the production of secondary metabolites was uniformly concentration-dependent in both solution types. *C. sativa* root exposure to increasing NaCl concentration solution decreased cannabinoid concentration in a linear fashion from 1 to 40 mM NaCl. Total THC equivalent (Δ^9^-THC + Δ^9^-THCA x 0.877), total CBD equivalent (CBD + CBDA x 0.877), Δ^9^-THCA, CBDA, CBGA and CBCA decreased linearly in response to increasing NaCl concentrations in plants grown in aquaponic and hydroponic solutions (**Figures 5A–F** ). Total cannabinoid concentration also decreased linearly in response to increasing NaCl concentrations in plants grown in aquaponic (% = 10.3 - 0.07 x NaCl (mM); R^2^ = 0.586; *P* = 0.0009) and hydroponic solution (% = 9.9 - 0.09 x NaCl (mM); R^2^ = 0.539; *P* = 0.0018). The rate of decrease in cannabinoid concentration for increase in NaCl concentration was higher for plants grown in hydroponic solution (-0.043%, -0.030%, -0.002%, -0.0016%·mM NaCl^-1^ for total CBD, total THC, CBCA and CBGA, respectively) compared to plants grown in aquaponic solutions (-0.035%, -0.022%, -0.001%, -0.0015%·mM NaCl^-1^ for total CBD, total THC CBCA and CBGA, respectively), for all cannabinoid relationships. Interestingly, Δ^9^-THC and CBD concentrations increased with increasing NaCl concentrations in plants grown in hydroponic solution, while no correlations to these cannabinoids were found in plants grown in aquaponic solution (**Figures 6A, B****)**. When Δ^9^-THC and CBD concentrations in hydroponic plants were expressed as content however, they did not increase with increasing NaCl concentration. For example, hydroponic plants in 1 mM NaCl solution had an average CBD and Δ^9^-THC content of 1.39 and 2.71 g·plant^-1^, respectively; while plants in 40 mM NaCl solution had 0.77 and 1.64 g·plant^-1^, respectively.

### Solution Dynamics

Between 14 and 28 DFS, both aquaponic and hydroponic solutions had an increasing pH·day^-1^ of 0.20 ± 0.015 and 0.18 ± 0.013, respectively. Between 30 and 63 DFS aquaponic and hydroponic solutions had a decreasing pH·day^-1^ of 0.08 ± 0.001 and of 0.06 ± 0.010 mS·cm^-1^, respectively. Both aquaponic and hydroponic solution had an increasing EC per day of 0.12 ± 0.008 and 0.13 ± 0.008 mS·cm^-1^, respectively, over the duration of the experiment. Plants grown in hydroponic solution had decreasing solution removal·day^-1^ with increasing NaCl concentrations when data was pooled over time (L·day^-1^ = 0.45 – 0.0044 x NaCl (mM); R^2^ = 0.350; *P* = 0.0007). Plants grown in aquaponic solution removed approximately 0.39 ± 0.010 L of solution per day. Water use efficiency had a negative correlation with NaCl concentration in both aquaponic (r = -0.37, *P* = 0.038) and hydroponic (r = -0.68, *P* < 0.0001) solutions.

At 35 DFS, after three days of exposure to plant roots, increasing NaCl concentrations in aquaponic solution had a negative linear relationship with NH_4_ removal (NH_4_ (mg) = 44.1 – 0.68 x NaCl (mM); R^2^ = 0.518; *P* = 0.0037); Mg removal [Mg (mg) = 11.8 – 0.67 x NaCl (mM); R^2^ = 0.370; *P* = 0.0209]; Zn removal [Zn (mg) = 0.38 – 0. 0.112 x NaCl (mM); R^2^ = 0.382; *P* = 0.0184] and Si removal [Si (mg) = 7.1 – 0.14 x NaCl (mM); R^2^ = 0.439; *P* = 0.0098]. There were no other significant differences calculated for absolute nutrient removal for any of the nutrients across solution and NaCl combinations at 35 DFS.

## Discussion

For the first time concentration-dependent ecophysiological responses to NaCl in a drug-type cultivar of *C. sativa* were described. In hydroponics, NaCl at a concentration of 40 mM resulted in phytotoxicity, evident through reticent growth and physiology. The negative effects of NaCl on reproductive parameters was evident at a much lower concentration, as yield and potency began declining at NaCl concentrations of 5 mM. Significant associations were found between leaf-level physiological performance and biomass production, suggesting impaired photosynthesis is one mechanism responsible, at least in part, for yield declines in high salinity. Notably, plants in the aquaponic solution had a certain NaCl tolerance, whereby the only effects of NaCl were the reduction of cannabinoid concentrations.

### NaCl Affects Physiology Through Osmotic Stress

The decrease in photosynthetic parameters from NaCl stress have been well reported in several other horticulture crops grown in soilless conditions, such as *Rosa × hybrida* L. (rose) (Cai et al., 2014) and *Lycopersicon esculentum* (tomato) (Lovelli et al., 2012); but never before in *C. sativa*. Presently, it was found that NaCl reduces leaf-level gas exchange at a concentration of 40 mM in hydroponic solution. A lesser photosynthetic sensitivity to NaCl has been found in zucchini which had decreased A at 5 mM compared A at 0.7 mM (Neocleous and Savvas, 2017) and in six different rose cultivars, which had reduced *g_s_* when grown with a 4.0 mS·cm^-1^ solution compared to a control (EC equivalent to 20 mM NaCl treatment) (Cai et al., 2014). The reduction in photosynthetic parameters, paired with decreasing solution uptake from plants grown in increasing NaCl concentrated hydroponic solution (demonstrated here), confirms that osmotic salt stress was likely impacting photosynthesis. The reduction in solution uptake from increased NaCl has also been reported in *Cucumis melo* L. (melon) (Neocleous and Savvas, 2016) and the reduction of osmotic potential in leaf tissue from NaCl stress has been observed in *Solanum melongena* L. (eggplant) (Shaheen et al., 2013). Furthermore, salt stress has been shown to create reactive oxygen species in glycophytes which can cause oxidative damage in leaves (Khare et al., 2015; Cheng et al., 2016).

Oxidative stress can damage photosynthetic enzymes, which can decrease the efficiency of photosystem II (Fv/Fm). In the present study, Fv/Fm was reticent: values found to be lower than 0.75 in the 20 and 40 mM NaCl treatments [i.e., significantly lower than a plant considered healthy (0.83) according to Johnson et al. (1993)]. The reduction of Fv/Fm induced by salt stress has been shown in other crops but to a lower extent than presently found. For example, solution with an EC of 8.0 mS·cm^-1^ (EC equivalent to NaCl concentration beyond what was used presently) caused only a 1.1% reduction in the Fv/Fm in roses (Cai et al., 2014) and solution with 60 mM NaCl caused only a 11.8% decrease in Fv/Fm (Fv/Fm = 0.75) in sesame compared to the control (Bazrafshan and Ehsanzadeh, 2014). Substantially lower Fv/Fm in the hydroponic 20 and 40 mM NaCl treatments was paired with visual observation of fully necrotic leaves and inflorescence tissues at 63 DFS. Such severe NaCl effects in the 20 and 40 mM hydroponic treatment may be attributed to inhibition of enzyme activity and eventual cell death (Munns, 2002). Fv/Fm may have also been slightly lower in all plants due to root rot, which was mildly exhibited at 21 DFS for all plants. The relationships between NaCl concentration and Fv/Fm suggests that *C. sativa* grown in hydroponic solution has less adaptive mechanisms to tolerate salinity compared to their near-descendent hemp-type cultivar, which have shown to partially tolerate NaCl in their aboveground tissues when grown in NaCl concentrations as high as 100 mM and germinated in 150 mM (Hu et al., 2018; Hu et al., 2019). Previous hemp research has suggested that NaCl stress can be tolerated through aquaporins in increased bast fiber development—as indicated by increased length in hypocotyl and radicles in NaCl stressed plants (Hu et al., 2018; Guerriero et al., 2019). Given the decrease in physiological parameters for plants grown in hydroponic solution, it is suggested that such mechanisms observed in hemp may not occur to the same extent in drug-type cultivars; however, further research is needed to quantify the production of radicals and stress related proteins to verify this. Furthermore, future research should involve multiple cultivars, to clarify if drug-type *C. sativa* cultivars have a cultivar-dependent response to NaCl as prominently shown in hemp-type cultivars (Liu et al., 2016).

Salt stress can also come in the form of Na^+^ accumulation in the leaves and nutrient antagonisms in the rootzone environment, particularly with K^+^. Glycophytes are unable to exclude salt at the roots, this allows ions to enter the plants and potentially accumulate in the leaves through transpiration forces. For example, Shaheen et al. (2013) found that NaCl at 50 mM and higher, caused a decrease in leaf K^+^ and an increase in leaf Na^+^ in eggplant. NaCl has also been shown to reduce leaf Mg^2+^ (Erdei and Kuiper, 1979). Since Mg^2+^ is an essential element in chlorophyll, such a displacement may have reduced Fv/Fm and other photosynthetic parameters. NaCl may have had a similar effect on leaf nutrition on drug-type *C. sativa*, given that hemp-type *C. sativa* is a glycophyte and a potential bio-accumulator. This has been demonstrated in multiple studies, as: Linger et al. (2002) found hemp to be a partial heavy metal accumulator, Landi (1997), found hemp to have different leaf nutrient concentrations based on the nutrients in the soil they were grown in and Ahmad et al. (2015) found that hemp accumulated Cd in different concentrations based on its rootzone nutrition. Leaf scorching (the chlorosis of leaf tips and margins) was observed at 42 DFS in most plants at NaCl treatments of 5 mM NaCl or higher. Although this observation has been attributed to K deficiency (Cockson et al., 2019), leaves were not sampled in the present study due to the limited leaf material available. Future studies should identify if NaCl affects Na^+^/K^+^/Mg^2+^ assimilation and radical oxidative species in *C. sativa* leaves, as this may have also been inducing stress. Furthermore, this research may determine if NaCl stress can be alleviated through nutrient additions; for example, it has been shown that *Capsicum annuum* (pepper) supplemented with K^+^ can alleviate some of the effects of NaCl (Rubio et al., 2010). It would also be interesting to see the effects of other neutral salts against NaCl, to examine if Na^+^ and Cl^-^ have individual oxidative effects as demonstrated in rice by Khare et al. (2015). Although most aquaponic plant parameters did not share negative relationships with NaCl concentrations, oxidative stress or K^+^ inhibition may still have occurred, especially given the negative relationship between NaCl and cannabinoid concentrations beginning at 5 mM.

### NaCl Decreases Yield and Potency

NaCl exceeding 5 mM resulted in decreased potency in plants grown in both solutions and decreased yield in plants grown in hydroponic solution. The decrease in photosynthetic ability caused by cumulative salt stress is suggested to contribute to the linear decrease in dry inflorescence biomass production, through decreased ability to assimilate carbon. This was reinforced through positive correlations between physiological parameters and inflorescence biomass in hydroponic plants. Although not previously reported in *C. sativa*, the linear decrease between inflorescence biomass and salinity have been reported for six flowering crops grown in soilless conditions with a similar NaCl range used presently (Sonneveld et al., 1999). The reduction in A, *g_s_*, Fv/Fm, and cannabinoid acid concentrations provides evidence that cumulative stress can cause a reduction in *C. sativa* physiology. This finding is important for *C. sativa* cultivators to consider, given many cultivators are interested in increasing cannabinoid concentrations through controlled stress applications to improve the highly economically and medically valuable inflorescence. It has been shown that cannabinoid concentrations can be increased through controlled drought stress application (Caplan et al., 2019). Caplan et al. (2019) increased THCA and CBDA concentrations by 12 and 13%, respectively, compared to the control, by applying a drought stress that induced a 50% decrease in leaf angle from the turgid state at 39 DFS and then allowing the plant to recover. Given that drought and salt can induce stress on plants through similar functions (Munns, 2002), the opposing results between our study and Caplan et al. (2019) is likely a result of stress being applied in a cumulative or perpetual fashion versus a periodic or single occurrence. An initial stress event can cause hormonal changes in the plant which can increase secondary metabolite production (Krasensky and Jonak, 2012). Metabolites such as proline, glutamine, asparagine, and soluble sugars often accumulate in plants under salt stress and may further regulate plant responses to environmental stresses while also impacting the quality of yield (Sanchez et al., 2011; Krasensky and Jonak, 2012). However, prolonged stress (evident at ≥ 5 mM NaCl here) can cause an increase in oxidative species in the plant, causing enzymes to malfunction and plant performance to decrease, as suggested in this study. Future research should investigate the effects of a single salt stress period, followed by a recovery period in an attempt to increase cannabinoid concentrations without sacrificing yield. Currently, *C. sativa* cultivators relying on recirculating solution should keep NaCl concentrations below 5 mM to prevent a reduction of yield and potency. Opposite to the decrease in cannabinoid acid concentrations, an increase in the concentrations of decarboxylated cannabinoids (THC and CBD) was observed in the dry inflorescence biomass of plants grown in hydroponic solution with increasing NaCl. The lack of natural cooling caused by osmotic pressure in the leaf, paired with the high radiation provided by the lights, may have mimicked and accelerate “drying effect”, causing an increase in decarboxylation of THCA to THC and CBDA to CBD, particularly in the 40 mM treatment where plants had necrotic inflorescence tissue (Tatsuo et al., 1967; Kimura and Okamoto, 1970).

### Potential Salt Tolerance in Aquaponics

Negative effects of NaCl on plant growth and physiology was evident in plants grown in hydroponic solution but not in plants grown in aquaponic solution. Varying NaCl effects based on solution was also reinforced through the death of four out of the six plants in the hydroponic 40 mM NaCl treatment, occurring at 47, 57, 60, and 61 DFS (deceased plants were still used for inflorescence biomass measurements), but none in the aquaponic solution. Furthermore, aquaponic plants in the 4 mM NaCl treatment had a higher dry inflorescence biomass than hydroponic plants in the 1 mM NaCl treatment. This indicates that aquaponics can be competitive with hydroponic production when supplemented with nutrients. Despite Na^+^ being suggested as a potential limitation in coupled aquaponic systems, this study shows that Na^+^ may not be a major limitation in deep water culture aquaponics (Yep, 2020). It had been previously shown that NaCl at concentrations as high as 34 mM in aquaponic solution did not have adverse effects on lettuce growth (Beauchamp et al., 2018); however, this had not been shown in *C. sativa*. Although the effects of NaCl in aquaponic solution contradicts what would be expected of a suboptimal nutrient solution, there is scarce research investigating NaCl in aquaponic solution and therefore our result is not contested. There are a few hypothetical reasons for *C. sativa* to have NaCl tolerance in aquaponic solution, however, further plant tissue and solution nutrient analyses are necessary to test these speculations.

Although both solutions had similar ECs, hydroponic solution had higher concentrations of N, P, K, and micronutrients, while aquaponic solution had higher concentrations of Si, SO_4_^-2^ and Mg^2+^. Recent research has identified Si as an important molecule in tolerating salt stress by regulating osmoticum in bast-fibers (Guerriero et al., 2016; Guerriero et al., 2019). The higher concentrations of Si in aquaponic solution (0.13 mM), compared to hydroponic solution (0.02 mM), may have allowed for greater Si to be assimilated, however plant nutrient tissue analysis was not conducted and therefore assimilation could not be confirmed. The high concentrations on P and low concentrations of Mg in the hydroponic solution also diverge from the standard Hoagland solution and may have adversely affected hydroponic plants compared to aquaponic plants, however, specific P and Mg concentration effects on *C. sativa* are unknown and require future investigation. The greatest macronutrient stoichiometry differences between solutions is the N:Mg and K:Mg ratios, which is 3.8 and 4.9 times greater in the hydroponic treatments compared to aquaponic treatments, respectively (**Table 1**). Such a difference may suggest that Mg was more limiting in the hydroponic treatment compared to the aquaponic solution which may relate to decreased Fv/Fm values; however, further investigation with tissue nutrient analysis is necessary to evaluate this. With aquaponics previously shown to have a rich population of PGPM (Schmautz et al., 2017; Bartelme et al., 2018), it is also possible that salt tolerance inducing PGPMs species such as *Bacillus*, *Azobacter*, and *Pseudomonas* strains were alleviating salt stress (Ilangumaran and Smith, 2017); however, future research is needed to identify these PGPM species in the growing solution to determine if such an effect is possible. Finally, NaCl tolerance may also be attributed to the increased organic particles in aquaponic solution. Coupled aquaponic systems relies on a constant stream of organic particles (suspended solids), that partially mineralize in the solution environment over time, releasing ions that may not be accounted for in chemical analyses (Rakocy, 2012; Goddek et al., 2018). This may be providing some additional osmoticum other than Na^+^, such as K^+^, as well as organic acids, hormones, and other metabolites, that could potentially have effects on plant response to NaCl stress (Rakocy, 2012; Ilangumaran and Smith, 2017). Additionally, the organic particles themselves, may be reducing the positive pressure generated by Na^+^ through accumulating on the rhizosphere, forming relationships with exudates and forming negative pressure (Asadi et al., 2009; Vives-Peris et al., 2020).

Given that the rootzone environment can have an effect on the results of an experiment, as demonstrated by Yep (2020) and evident through solution type here, this experiment should be repeated in more common commercial *C. sativa* rootzone systems (i.e., mineral wool with drip irrigation) comprising different aqueous, solid, and gaseous fractions to better understand the effects of NaCl. Future *C. sativa* NaCl experiments should also investigate multiple drug-type *C. sativa* cultivars given that NaCl effects have been shown to vary across food crop cultivars (Kong et al., 2014) and hemp cultivars (Hu et al., 2019).

## Conclusion

*C. sativa* yield and potency decreased with perpetual root-exposure to increasing NaCl concentrations, with 40 mM proving to be phytotoxic for plants grown in hydroponic solution. Solution culture *C. sativa* cultivators should be cautious in using NaCl ≥ 5 mM in their fertigation solution, particularly those that recirculate hydroponic solution. By measuring Na^+^ and Cl^-^ concentrations in their source water, fertilizers, and recirculating nutrient solution, cultivators can now make more informed decisions as to when their fertigation solution should be replaced. This can reduce potential decreased *C. sativa* potency and yield caused by salt stress, while also mitigating unnecessary solution discharge. The specific negative threshold of NaCl stress on *C. sativa*, between 1 and 10 mM, as well the effects of a periodic salt stress is worthy of further investigation. Alternatively, our research has identified that a certain aspect of aquaponic solution may allow resistance to NaCl stress. Future research investigating what specific mechanisms at the rhizosphere that may allow this in aquaponic solution, could be extrapolated to hydroponic production, thus allowing for optimal growth with higher NaCl concentrations. This would effectively decrease fertigation solution discharge and may also reduce the filtration needs associated with removing Na^+^, which can be costly and energy intensive.

## Data Availability Statement

The original contributions presented in the study are included in the article/supplementary material; further inquiries can be directed to the corresponding author.

## Author Contributions

BY, NG, and YZ designed the experiment. YZ is the PI and supervised the research. BY carried out the experiment, performed statistics and manuscript writing. BY, NG, and YZ finalized the manuscript.

## Funding

This work was funded by the Natural Science and Engineering Research Council of Canada (Grant # 533527-18) and Green Relief Inc.

## Conflict of Interest

The authors declare that this study received funding from Green Relief Inc. The funder was not involved in the study design, collection, analysis, interpretation of data, the writing of this article or the decision to submit it for publication.

## Abbreviations

A, Leaf net CO2 assimilation rate; DFS, Day(s) in the flowering stage; EC, electroconductivity; Fv/Fm, chlorophyll fluorescence; *g_s_*, Stomatal conductance; PAR, Photosynthetically active radiation; PGPM, Plant growth promoting microorganisms.
